# Antimicrobial peptides at work: interaction of myxinidin and its mutant WMR with lipid bilayers mimicking the *P. aeruginosa* and *E. coli* membranes

**DOI:** 10.1038/srep44425

**Published:** 2017-03-15

**Authors:** Lucia Lombardi, Marco Ignazio Stellato, Rosario Oliva, Annarita Falanga, Massimiliano Galdiero, Luigi Petraccone, Geradino D’Errico, Augusta De Santis, Stefania Galdiero, Pompea Del Vecchio

**Affiliations:** 1 Department of Experimental Medicine, Università della Campania “Luigi Vanvitelli”, via De Crecchio, 80134 Naples, Italy; 2 Department of Chemical Sciences, University of Naples “Federico II”, via Cintia, 80126 Naples, Italy; 3 Department of Pharmacy, University of Naples “Federico II”, Via Mezzocannone 16, 80134 Naples, Italy

## Abstract

Antimicrobial peptides are promising candidates as future therapeutics in order to face the problem of antibiotic resistance caused by pathogenic bacteria. Myxinidin is a peptide derived from the hagfish mucus displaying activity against a broad range of bacteria. We have focused our studies on the physico-chemical characterization of the interaction of myxinidin and its mutant WMR, which contains a tryptophan residue at the N-terminus and four additional positive charges, with two model biological membranes (DOPE/DOPG 80/20 and DOPE/DOPG/CL 65/23/12), mimicking respectively *Escherichia coli* and *Pseudomonas aeruginosa* membrane bilayers. All our results have coherently shown that, although both myxinidin and WMR interact with the two membranes, their effect on membrane microstructure and stability are different. We further have shown that the presence of cardiolipin plays a key role in the WMR-membrane interaction. Particularly, WMR drastically perturbs the DOPE/DOPG/CL membrane stability inducing a segregation of anionic lipids. On the contrary, myxinidin is not able to significantly perturb the DOPE/DOPG/CL bilayer whereas interacts better with the DOPE/DOPG bilayer causing a significant perturbing effect of the lipid acyl chains. These findings are fully consistent with the reported greater antimicrobial activity of WMR against *P. aeruginosa* compared with myxinidin.

The rise in antibiotic resistance caused by pathogenic bacteria has compelled the challenging search for new antibiotic therapeutic agents such as antimicrobial peptides (AMPs). AMPs are an essential part of the innate immune response and their ubiquitous presence in nature (microorganisms, insects, invertebrates, amphibians, plants, birds, and mammals) attests their key role in building up the defense strategies of almost every organism^1^,^2^,^3^,^4^. Most known AMPs are small peptides, formed of 12 to 60 amino acids with molecular masses <10 kDa^5^. Recently, several AMPs have been identified in the marine environment, which act as the first line of defense against a broad spectrum of pathogens^6^. Among AMPs discovered in fish, there is the peptide myxinidin, identified from the epidermal mucus of hagfish (*Myxine glutinosa L*.) by Subramanian *et al*.^7^. Myxinidin is one of the shortest AMPs discovered so far (12-amino-acid peptide GIHDILKYGKPS-NH_2_ with a net charge of +2) and showed potent antibacterial activity against a wide range of bacteria and yeast pathogens^7^. Cantisani *et al*. designed a series of structurally modified synthetic myxinidin analogs and determined their antimicrobial activities to elucidate the structure-function relations and the effect of each amino acid residue on the antimicrobial activity^8^,^9^.

The results suggest that one of the myxinidin mutant, named WMR (13 amino acid peptide **W**GI**RR**ILKYGK**R**S-NH_2_ with a net charge of +6), has a higher antimicrobial activity than myxinidin against *Gram positive* and *Gram negative* bacteria^8^,^9^. In particular, WMR contains a tryptophan residue at the N-terminus; this amino acid residue has been usually found to interact with the interfacial region of membranes and presents strong membrane-disruptive activity^10^,^11^. Furthermore, WMR contains a higher number of positively charged amino-acids (arginines) compared to the native sequence, which are essential for the initial attraction between AMPs and the negatively charged phosphate groups of the bio-membrane.

Many different mechanisms of membrane damage have been proposed for AMPs, depending on their physico-chemical properties and on the target bio-membranes^12^,^13^,^14^,^15^. As a matter of fact, AMPs interact selectively with prokaryotic cells and this behavior is believed to be a consequence of the difference in the chemical composition between prokaryotic and eukaryotic membranes^16^. In fact, bacterial membranes contain a high percentage of negatively charged phospholipids, while eukaryotic membranes mainly contain zwitterionic phospholipids. This selectivity for prokaryotic membranes is a key element to distinguish between antibacterial and toxic molecules as demonstrated with cecropins^17^, magainins^18^, dermaseptins^19^ and other AMPs^20^.

The mechanism of the final killing step of AMPs will depend very strongly on a range of physico-chemical properties such as peptide concentration and type^13^ as well as secondary structure adopted in the presence of the membrane environments^21^,^22^,^23^. Thus, understanding how lipid composition affects the membrane biophysical properties and modulates its interaction with AMPs represents the basis for understanding AMPs selectivity for bacterial bio-membranes.

The major components of bacterial membranes are zwitterionic phosphatidylethanolamine (PE), anionic phosphatidylglycerol (PG) and cardiolipin (CL). Cardiolipin is an unusual anionic phospholipid found in the plasma membranes of many types of *Gram-negative* and *Gram-positive* bacteria and in the mitochondrial and chloroplast inner membranes of eukaryotes^24^,^25^. This phospholipid is a relatively small component of the total membrane lipid composition but plays a key role in the dynamics of bio-membranes^25^. Despite the biochemical and biomedical importance of this lipid, relatively few studies of the organization of CL bilayers have been performed. Interestingly, the analysis of the antimicrobial activity data has shown that myxinidin is less efficient compared to WMR against bacteria containing higher content of CL in their membranes^9^. In order to understand the molecular basis of these differences in the antimicrobial activity of these two peptides, we focus our attention on the mode of interaction between myxinidin and its mutant WMR with two different model bio-membranes, composed by DOPE/DOPG (80/20% mol) and DOPE/DOPG/CL (65/23/12% mol), mimicking respectively *Escherichia coli* and *Pseudomonas aeruginosa*^8^. Moreover, in order to evaluate the effect of the charge and CL presence we tested other liposomes composed by DOPE/DOPG (53/47% mol) which contain the same number of negative charges of DOPE/DOPG/CL (65/23/12% mol). In particular, we combined calorimetric, Isothermal Titration Calorimetry and Differential Scanning Calorimetry (ITC and DSC) and spectroscopic, Fluorescence and Electron Paramagnetic Resonance (EPR) techniques together with other methodologies, Dynamic Light Scattering (DLS) and Surface Plasmon Resonance (SPR) providing a comprehensive and detailed analysis of the peptides-membrane interactions. All our data clearly show that the presence of CL lipid (and not just the membranes charge) plays a key role in the WMR-membrane interaction. Our results are fully consistent with the reported greater antimicrobial activity of WMR against *P. aeruginosa* compared with myxinidin and allow correlating the different antimicrobial activity of these two peptides with a distinct behavior at the level of peptide-lipid interactions.

## Methods

### Chemicals

The 9-fluorenylmethoxycarbonyl (Fmoc)-protected amino acids used for the peptide synthesis, the Rink amide MBHA resin (0.54 mmol/g) and the activators N-hydroxybenzotriazole (HOBT) and O-benzotriazole-N,N,N’,N’-tetramethyl-uronium-hexafluoro-phosphate (HBTU) were purchased from Novabiochem. HPLC-grade acetonitrile (ACN), trifluoroacetic acid (TFA), dry N,N-dimethylformamide (DMF) and N-methylpirrolidone (NMP) were purchased by Romil Del Chimica. All other reagents solid-phase peptide synthesis (piperidine, diisopropilammina (DIPEA)) were purchased from Sigma-Aldrich. Liquid chromatography-mass spectrometry (LC-MS) analyses were performed on a Thermo Finnigan LC-MS with an electrospray source (DECA) on a Phenomenex Jupiter 4 μm Proteo C12 90 Å 150 by 4.6 mm column. Purification was carried out on a Phenomenex Jupiter 10 μm Proteo 90 Å 250 by 21.20 mm column. Phospholipids: 1,2-dioleoyl-sn-glycero-3-phosphoethanolamine (DOPE), 1,2-dioleoyl-sn-glycero-3-phospho-(1′-rac-glycerol) sodium salt (DOPG), 1,2-dipalmitoyl-sn-glycero-3-phosphoethanolamine (DPPE), 1,2-dipalmitoyl-sn-glycero-3-phospho-(1′-rac-glycerol) (sodium salt) (DPPG) and cardiolipin (CL) sodium salt (Heart, Bovine) the fluorescent probes N-(7-nitro-benz-2-oxa-1,3-diazol-4-yl) phosphatidylethanolamine (NBD-PE) and N-(Lissaminerhodamine-Bsulfonyl) phosphatidylethanolamine (Rho-PE), as well as the spin labels 1-palmitoyl-2-stearoyl-(5-doxyl)-sn-glycero-3-phosphocholine and 1-palmitoyl-2-stearoyl-(14-doxyl)-sn-glycero-3-phosphocholine (5-PCSL and 14-PCSL) were purchased from Avanti Polar Lipids (Birmingham, AL, USA), Phosphate-buffered saline (PBS) tablets were bought by Life Technologies Corporation.

8-aminonaphtalene-1,3,6-trisulfonic acid, disodium salt (ANTS) and p-xylene-bis-pyridinium bromide (DPX) were purchased from Molecular Probes. Triton X-100 was purchased by Sigma. Deionized water was used for the buffer solutions and sample preparation.

### Peptide synthesis

Peptides were synthesized using the standard solid phase 9-fluorenylmethoxy carbonyl (Fmoc) method and purified as previously reported^9^. Briefly, 100 μmol of each peptides were synthesized on a Rink amide MBHA (0.54 mmol/g) resin by consecutive deprotection (30% v/v piperidine in DMF, twice for 10 min) and coupling (4 equivalents of amino acid plus 4 equivalents of 0.45 M HOBT/HBTU in DMF, and 8 equivalents of DIPEA 2 M in NMP, double coupling and 1 h for each coupling) cycles. Peptides were cleaved from the resin and deprotected by treatment with TFA/thioanisole/anisole/water/EDT 82.5/5/5/5/2.5 v/v. and precipitated in ice-cold ethylic ether. The peptide was dissolved in water and freeze-dried. Analysis of the crude peptides was performed by electrospray ionization (ESI) LC-MS using a linear gradient of acetonitrile (0.1% TFA) in water (0.1% TFA) from 5 to 70% in 15 min. Peptides were purified by preparative reversed-phase high-performance liquid chromatography (RP-HPLC) using a gradient of acetonitrile (0.1% TFA) in water (0.1% TFA) from 5 to 70% in 25 min. All purified peptides were obtained with good yields (90 to 95%).

### Liposome preparation

Liposomes of the appropriate size were prepared for different experiments. An appropriate amount of lipids was weighed and dissolved in CHCl_3_/CH_3_OH (70/30 v/v) mixture to prepare liposomes at different composition. Typical mixtures volumes were 2 mL of a lipid concentration range of 0.1–1 mM. In these conditions fully hydrated liposomes were obtained. A thin film of lipids was obtained through evaporation of the organic solvent with dry nitrogen gas and vacuum desiccation. Lipid films were kept in vacuum overnight to remove all residual organic solvent, then hydrated with a definite amount of PBS buffer pH 7.4 and finally vortexed to obtain a suspension of Multi Lamellar Vesicles (MLVs). In addition, Large Unilamellar Vesicles (LUVs) were prepared by extrusion using a Mini-Extruder (Avanti Polar Lipid Inc.) according to method described in Hope *et al*.^26^. In fact, the MLVs suspension was freeze-thawed six times then passed through a 100 nm pore size polycarbonate membrane 25 times and the obtained LUVs suspensions were used to study the binding with peptides. Dynamic light scattering measurements were performed to check the size of the vesicles after the extrusion protocol.

### Lipid mixing assays

Membrane lipid mixing was monitored using the fluorescence resonance energy transfer assay (FRET) reported by Struck *et al*.^27^. This assay exploits the dilution of the NBD-PE (donor) and Rho-PE (acceptor). Vesicles containing 0.6% mol of each probe were mixed with unlabelled vesicles at a 1:4 ratio in buffer 5 mM HEPES plus 100 mM NaCl pH 7.4 (final lipid concentration in the cuvette 0.1 mM). We monitored the change in donor emission as aliquots of peptides were added to vesicles at different peptide/lipid molar ratio. Dilution due to membrane mixing promoted by peptides results in an increase in NBD-PE fluorescence. All fluorescence measurements were carried out with a Cary Eclipse fluorescence spectrophotometer. The NBD emission at 530 nm was followed with the excitation wavelength set at 465 nm. A cut off filter at 515 nm was used between the sample and the emission monochromator to avoid scattering interferences. The fluorescence scale was calibrated such as 0% value corresponding to the initial residual fluorescence of the labeled vesicles and as 100% value corresponding to complete lipid mixing upon the addition of Triton X-100 (0.05% v/v). Lipid mixing experiments were repeated at least three times and results were averaged. All fluorescence measurements were conducted in DOPE/DOPG (80/20% mol) DOPE/DOPG/CL (65/23/12% mol) LUVs at 25 °C and DOPE/DOPG (53/47% mol).

### Inner-monolayer phospholipid mixing assay

Inner-monolayer phospholipid mixing assay was measured by a modification of the lipid mixing assay reported before. The unlabelled vesicles were prepared as done for lipid mixing assays. The labelled vesicles were prepared in a different buffer (10 mM TRIS, 100 mM NaCl, 1 mM EDTA, pH 7.4) and were treated with sodium dithionite 100 mM (from a stock solution of 200 mM dithionite in 200 mM TRIS, pH 10.0) to reduce completely the NBD-labelled phospholipid located at the outer monolayer of the membrane, for approximately 30 min on ice in the dark. Sodium dithionite was then removed by size exclusion chromatography through a Sephadex G-50 DNA Grade filtration column (GE Healthcare) eluted with a buffer containing 5 mM HEPES plus 100 mM NaCl pH 7.4. All fluorescence measurements were conducted in DOPE/DOPG (80/20% mol), DOPE/DOPG/CL (65/23/12% mol) and DOPE/DOPG (53/47% mol) LUVs and at 25 °C.

### ANTS/DPX leakage

The ANTS/DPX assay^28^ was used to measure the ability of peptides to induce leakage of ANTS/DPX pre-encapsulated in liposomes. Details of this assay can be found elsewhere^29^. The peptide, in a stock solution containing 5 mM HEPES and 100 mM NaCl pH 7.4, was added to vesicle suspensions (1 mM lipid) at different molar ratio. Lipid mixing experiments were repeated at least three times and results were averaged. All fluorescence measurements were conducted in DOPE/DOPG (80/20%mol), DOPE/DOPG/CL (65/23/12%mol), DOPE/DOPG (53/47% mol) LUVs at 25 °C.

### Dynamic Light Scattering

DLS measurements, at the temperature of 25 °C, were performed with a setup composed by a Photocor compact goniometer, a SMD 6000 Laser Quantum 50 mW light source operating at 5325 Å, a PMT and a correlator obtained from Correlator.com. All the experiments were performed at the scattering angle of 90° (θ). The value of the scattering vector q = 4πn/λsin(θ/2) was calculated assuming the refractive index of the water suspension of n = 1.33. The scattered intensity correlation function was analyzed using a regularization algorithm^30^. The measured diffusion coefficients were taken as the z-averaged diffusion coefficients of the obtained distributions. For spheres diffusing in a continuum medium at infinite dilution, the diffusion coefficient 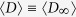 is dependent on the sphere radius R_h_ (hydrodynamic radius) through the Stokes–Einstein equation:


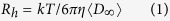


where T is the absolute temperature, k is the Boltzmann constant and η is the medium viscosity (assumed to be 0.89 cP for the water suspension). For non spherical particles, R_h_ represents the radius of equivalent spherical aggregates. In this hypothesis, Eq. 1 can be reasonably used to estimate the average hydrodynamic radius of the aggregates.

To obtain the R_h_ values at least three measurements of the diffusion coefficients of the aggregates for each analyzed samples were performed. For both lipid systems under consideration, LUVs were used at the same concentration of ITC experiments (0.2 mM) in the absence and presence of the two peptides.

### Electron Paramagnetic Resonance

EPR spectra of the spin labels 5-PCSL or 14-PCSL (1% by weight over total lipids) in DOPE/DOPG/CL (65/23/12% mol) and DOPE/DOPG (80/20% mol) MLVs as aqueous suspensions were recorded on a Elexys E-500 EPR spectrometer from Bruker (Rheinstetten, Germany) operating in the X band. The same lipid systems were investigated in the presence of variable amounts of myxinidin or WMR peptides. Capillaries containing the samples were placed in a standard 4 mm quartz sample tube. The temperature of the sample was regulated and maintained constant at 25 °C. The instrumental settings were as follows: sweep width, 120 G; resolution, 1024 points; modulation frequency, 100 kHz; modulation amplitude, 1.0 G; time constant, 20.5 ms, incident power, 5.0 mW. Several scans, typically 16, were accumulated to improve the signal-to-noise ratio.

### Differential Scanning Calorimetry

DSC measurements were performed using a nano-DSC from TA instruments (New Castle, DE, USA). For all DSC experiments, MLVs were used. The excess molar heat capacity function, <ΔC_p_> was obtained after a baseline subtraction, assuming that the baseline is given by the linear temperature dependence of the pre-transition heat capacity. A buffer-buffer scan was subtracted from the sample scan. For DSC experiments MLVs of DPPE and DPPG phospholipids were prepared. The lipid films of two model bio-membranes were prepared as described above, then hydrated using PBS buffer, warmed at a temperature above the transition temperature of liposomes (70 °C) for 10 min and vortexed to obtain a homogeneous dispersion. A volume of 300 μL of lipids mixtures (0.5 mM) in the absence or in the presence of myxinidin and WMR peptides at different peptide/lipid ratios was placed in the calorimetry vessel. Successive heating and cooling scans were performed for each sample operating at a scan rate of 1 °C min^−1^ over the temperature range of 25–80 °C. Samples were prepared immediately before the DSC experiment, by adding the appropriate amount of peptide solution to the vesicles suspension and waiting 20 min to ensure that equilibrium has been reached. Successive heating and cooling scans are superimposed, confirming the reversibility of the process. DSC data were analyzed by means of the NanoAnalyze software supplied with the instrument and plotted using the Origin software package.

### Isothermal Titration Calorimetry

ITC measurements were performed using a nano-ITC III (TA instruments, New Castle, DE, USA) at 25 °C. The peptide solution (50–100 μM) was injected in the calorimetric vessel (1 mL) containing a lipid dispersion (0.2–0.5 mM concentration range). Under these conditions, the lipid is much in excess over the peptide during the whole titration experiment, and the injected peptide is completely bound to the membrane surface. Each injection should produce the same heat providing the binding enthalpy when divided by the mole of peptide. The peptide solution was injected in aliquots of 10 μL with 400 s intervals between the individual injections.

### Surface Plasmon Resonance

SPR experiments were carried out with a BIAcore 3000 analytical system (Biacore, Uppsala, Sweden) using the HPA and the L1 sensor chip. The HPA sensor chip contains hydrophobic alkanethiol chains, which are covalently bound to a gold surface, then a lipid heteromonolayer can be created by introducing liposomes to the chip; while the L1 sensor chip contains hydrophobic alkanethiol chains with exposed polar headgroups, and a lipid bilayer can be created. The complete coverage of the surface with a polar lipid monolayer generates a membrane-like environment where analytes in aqueous buffer interact with it. The used experimental protocol was previously described by Mozsolits *et al*.^31^. The operating temperature was 25 °C. All solutions were freshly prepared, degassed, and filtered through 0.22 μm pores. After cleaning as indicated by the manufacturers, the BIAcore 3000 instrument was left running overnight using Milli-Q water as eluent to thoroughly wash all liquid-handling parts of the instrument. The chip was then installed, and the alkanethiol surface was cleaned by an injection of the nonionic detergent N-octyl β-D-glucopyranoside (25 μl, 40 mM) at a flow rate of 5 μl/min. DOPE/DOPG/CL (65/23/12%mol) and DOPE/DOPG (80/20% mol) SUVs (80 μl, 0.5 mM) were then applied to the chip surface at a flow rate of 2 μl/min. NaOH 10 mM and a flow rate to 50 μl/min were used to remove any multilamellar structures from the lipid surface, which resulted in a stable baseline corresponding to the lipid monolayer linked to the chip surface. The negative control albumin bovine serum (BSA) was injected (25 μl, 0.1 mg/ml in PBS) to confirm complete coverage of the nonspecific binding sites.

WMR solutions and myxinidin solutions (30 μl at a flow rate of 5 μl/min) were injected onto the lipid surfaces followed by PBS buffer alone for 15 min to allow peptide dissociation. Changes in the reflective index of the surface layer of peptide and lipids in contact with the sensor chip were then translated to response units. A sensorgram is obtained by plotting the SPR angle against time. Analysis of the interaction was performed from a series of sensorgrams obtained at different peptide concentrations which were analyzed by curve fitting using numerical integration analysis. The BIA evaluation software was used to perform complete kinetic analyses of the sensorgrams and the two-state reaction fitting model was used considering literature data on the binding mechanism of AMPs^15^,^32^. This model describes two reaction steps^31^ which, in terms of peptide-lipid interaction, may correspond to i) peptide (P) binds to lipids (L) to give PL and ii) the complex PL changes to PL*, which cannot dissociate directly to P + L and which may correspond to partial insertion of the peptide into the lipid bilayer.


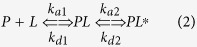


The corresponding differential rate equations for this reaction model are represented where RU_1_ and RU_2_ are the response units for the first and second steps, respectively, C_A_ is the peptide concentration, RU_max_ is the maximum peptide binding capacity (or equilibrium binding response), and k_a1_, k_d1_, k_a2_, and k_d2_ are the association and dissociation rate constants for the first and second steps, respectively:


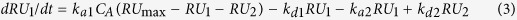



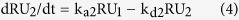


We used χ^2^ statistical tests to validate the model.

## Results and Discussion

### Lipid mixing assays

**Figure 1:**
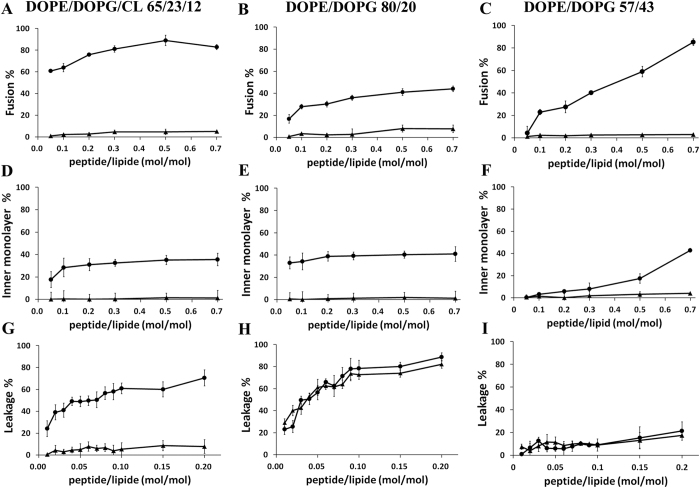
Fusion promoted by myxinidin (squares) and WMR (triangles) in DOPE/DOPG/CL (65/23/12% mol) (**A**), DOPE/DOPG (80/20% mol) (**B**) and DOPE/DOPG (57/43% mol) (**C**) liposomes; inner monolayer fusion promoted by myxinidin (squares) and WMR (triangles) in DOPE/DOPG/CL (65/23/12% mol) (**D**) and DOPE/DOPG (80/20% mol) (**E**) and DOPE/DOPG (57/43% mol) (**F**) liposomes; leakage promoted by myxinidin (squares) and WMR (triangles) in DOPE/DOPG/CL (**G**), DOPE/DOPG (80/20% mol) (**H**) and DOPE/DOPG (57/43% mol) (**I**) liposomes.

To investigate the fusogenicity of myxinidin and WMR in DOPE/DOPG (80/20% mol), DOPE/DOPG/CL (65/23/12% mol) and DOPE/DOPG (53/47% mol), FRET experiments were performed. A population of LUVs labeled with NBD-PE (donor) and Rho-PE (acceptor) was mixed with a population of unlabeled LUVs and increasing amounts of peptide were added. The peptide-induced membrane fusion resulted in a dilution of the fluorescent-labeled vesicles, hence de-quenching of the donor fluorescence. The dependence of the percentage of lipid mixing on the peptide to lipid molar ratio was analyzed. Figure 1 (panels A,B) shows the results of lipid mixing assays of both lipid systems for myxinidin and WMR at 25 °C. In contrast to myxinidin, the mutant peptide WMR induced significant levels of fusion in both types of LUVs, suggesting that it was able to interact with the bilayers destabilizing the membrane structures. In particular, we obtained a higher fusion activity when WMR was in the CL-containing vesicles. In Fig. 1 (panel C) lipid mixing is reported for LUVs composed of DOPE/DOPG (53/47% mol) in order to take into account the electrostatic contribution. We still do not observe any fusion with mixinidin, while for WMR we observe fusion but with a lower extent compared to LUVs containing CL suggesting that the charge could be not the only component responsible for the WMR-membrane interaction.

### Inner-monolayer phospholipid mixing assay

In the inner monolayer assay, the fluorescence from the outer monolayer of vesicles was eliminated by the addition of a reducing agent (dithionite), permitting to observe the extent of lipid mixing between the inner monolayers of vesicles in solution. Figure 1 (panels D,E,F) shows a significant fusion of the inner monolayer for WMR in all lipid systems. This is slightly lower than the fusion level obtained in the lipid mixing experiment, since the latter measures both hemi-fusion and complete fusion. Therefore, this assay clearly indicates that the peptide WMR is able to induce fusion of both the inner and the outer monolayers. For myxinidin, no inner monolayer fusion was observed.

### ANTS/DPX leakage

The effect of myxinidin and WMR on the release of encapsulated fluorophores in model membranes made of DOPE/DOPG (80/20% mol), DOPE/DOPG/CL (65/23/12% mol) and DOPE/DOPG (53/47% mol) LUVs has been studied. Release of ANTS and DPX from vesicles is commonly used as a measure of bilayer perturbation and interpreted as “transient pore formation”^29^. When ANTS (fluorophore) and DPX (quencher) were both in the internal watery part of liposomes, we did not observe ANTS fluorescence; but when pore formation occurred, the two molecules escaped from vesicles and drifted apart from each other resulting in an increase of ANTS fluorescence intensity. The leakage experiment (Fig. 1 panels G,H,I) shows that WMR exhibits a greater leakage than myxinidin with DOPE/DOPG/CL liposomes, but with DOPE/DOPG (80/20% mol) liposomes, the leakage promoted by the two peptides is comparable. Instead both WMR and myxinidin do not display leakage in DOPE/DOPG (53/47% mol) vesicles. The comparison of the results obtained with the DOPE/DOPG (53/47% mol) and DOPE/DOPG/CL (65/23/12% mol) liposomes (having the same charge), strongly suggest that CL plays a key role in the WMR-membrane interaction. On the contrary, the same comparison for myxinidin suggests that the presence of CL is not as specific as for WMR in determining the myxinidin-membrane interaction. On the other hand, the inability of myxinidin to induce leakage with the DOPE/DOPG (53/47% mol) is in sharp contrast with the result obtained on the DOPE/DOPG (80/20% mol). This result reveals that increasing the vesicle charge has a detrimental effect on the membrane-perturbing capability of myxinidin. A tentative explanation can be that the DOPE/DOPG (53/47% mol) not only does not contain the CL but has a too high content of negative charges which may struck the peptide on the surface of the membrane bilayer. In summary, the whole of the fluorescence-based experiments coherently show that WMR is able to significantly perturb all the studied liposomes whereas myxinidin has a major effect only on the less charged DOPE/DOPG (80/20% mol) liposomes. Further, differently from myxinidin, WMR has a preferential interaction with the CL-containing membrane. The liposome DOPE/DOPG (53/47% mol) was only used to confirm that the observed differences in the peptide-membrane interaction were not only due to electrostatic effects, thus we decided not to use this condition for further experiments but rather to focus the attention on the DOPE/DOPG (80/20% mol) liposomes mimicking the *E. coli* membrane.

### Dynamic Light Scattering

**Figure 2:**
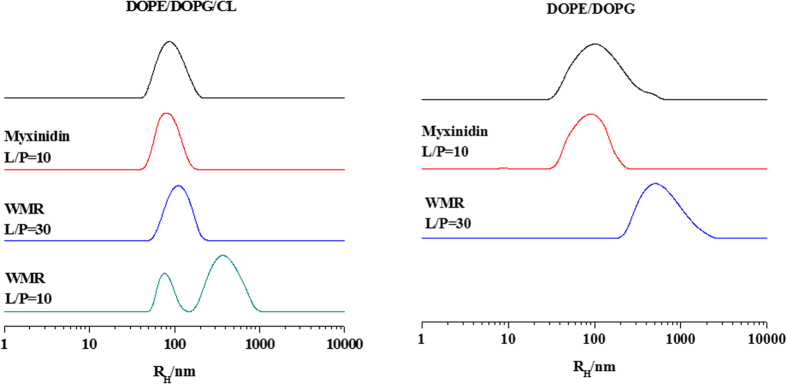
Hydrodynamic radius distribution functions of DOPE/DOPG/CL and DOPE/DOPG liposomes in the absence and presence of myxinidin and WMR peptides at the indicated lipid/peptide (L/P) ratio.

DLS experiments on DOPE/DOPG/CL and DOPE/DOPG LUVs suspension were performed in order to investigate the influence of peptides on liposome size and distribution. The hydrodynamic radius distribution functions for both lipid systems before and after the addition of myxinidin or WMR are reported in Fig. 2. The average diffusion coefficients of liposomes, and the corresponding hydrodynamic radii, are reported in Table 1. In the absence of any peptide, both DOPE/DOPG/CL and DOPE/DOPG lipid mixtures form a single population of LUVs with similar average dimension (as imposed by the membrane used for the extrusion procedure). DOPE/DOPG/CL LUVs present a narrower distribution. The hydrodynamic radius distributions show that both liposomes do not change upon the addition of myxinidin (red lines in Fig. 2), indicating that the unilamellar vescicles remain the predominant lipid aggregates even at L/P = 10 of myxinidin. For WMR peptide a change in the hydrodynamic radius distributions for both lipid systems is already observed at L/P = 30 (Blue lines Fig. 2). The variation extent depends on the lipid system. In fact, in the case of DOPE/DOPG/CL lipid mixtures, just a slight increase in the hydrodynamic radius is observed (from 88 ± 5 nm to 104 ± 2 nm), while for the DOPE/DOPG lipid mixtures, a dramatic increase in the liposomes dimensions (460 ± 80 nm) is observed, suggesting the formation of either multilamellar aggregates or liposome clusters. It is interesting to observe that, at this L/P ratio, peptide molecules positioned at the DOPE/DOPG liposome surface would almost completely neutralize its charge. With further increasing the WMR peptide concentration (up to L/P = 10) both types of vesicles undergo a structural rearrangement. The sample containing the DOPE/DOPG lipid vesicles shows a correlation function that do not tend to zero, indicating the formation of large aggregates whose diffusion is not Brownian (suggesting a macroscopic separation of a lipid phase). The CL-containing bilayer shows a bimodal distribution, which is usually associated to the co-existence of unilamellar and larger aggregates (multilamellar or clustered liposomes). The presence of large-size aggregates can be interpreted as due to a positioning of the peptide at the liposome surface (note that this L/P ratio corresponds to the neutralization of DOPE/DOPG/CL charges). Interestingly, in the case of DOPE/DOPG/CL lipid mixtures, unilamellar aggregates persist also at low L/P ratio, suggesting that the co-existence of an alternative peptide-membrane interaction mechanism, which is absent in the case of DOPE/DOPG liposomes. These results are consistent with the fluorescence results showing some degree of fusion for both membranes in the presence of WMR.

### Electron Paramagnetic Resonance

**Figure 3:**
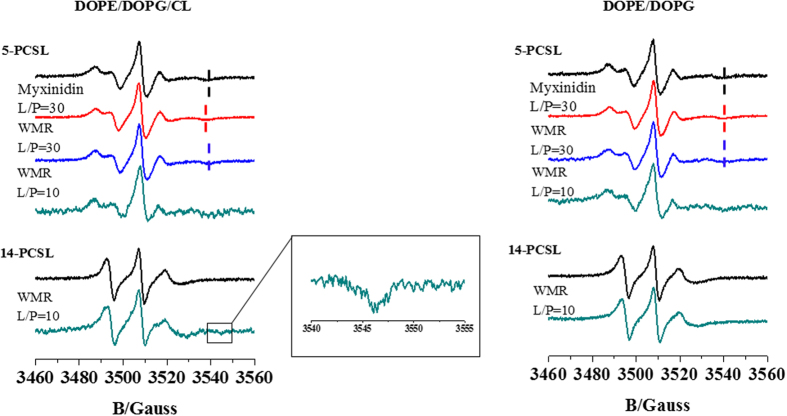
EPR spectra of 5- and 14-PCSL in the lipid systems investigated in this study, in the presence and absence of myxinidin and WMR peptides.

EPR spectra of different spin-labelled lipids inserted in DOPE/DOPG/CL and DOPE/DOPG liposomes were performed to monitor variations of the bilayer microstructure and dynamics due to the interaction with myxinidin or WMR. Two spin-labelled lipids were used: 5-PCSL, which presents the nitroxide reporter group close to the hydrophilic headgroup, and 14-PCSL, in which the reporter group is located close to the terminal methyl of the chain. 5-PCSL spectra furnish information on the bilayer region just underneath the interface with the aqueous medium, while 14-PCSL spectral features reflect the structural and dynamical status of bilayer inner core^33^. First, the EPR spectra in the absence of any peptide were acquired, (Fig. 3). For both lipid systems, 5-PCSL spectra present a well-defined axially anisotropic lineshape, as detectable from the splitting of the outer signals (solid-line spectra, upper and lower part of the figure for DOPE/DOPG/CL and DOPE/DOPG mixtures, respectively). This evidence is ascribable to the restriction of the nitroxide group motion due to its closeness with the interface-anchored headgroup, and is a typical feature of lipid bilayers. In contrast, three-line spectra are observed for 14-PCSL, indicative of an almost isotropic label motion. This ordering and dynamic gradient along the normal to the interface is typical of bilayer in the disordered liquid crystalline state^34^.

In order to quantitatively analyze the spectra, we determined the order parameter, S, which reflects the local ordering of the lipid tails around the acyl chain segment to which the paramagnetic label is bound, and the isotropic hyperfine coupling constant, a_N_’, which is an index of the local polarity^35^. Within the experimental uncertainty, these parameters present the same values in the two lipid systems, see Table 2, thus indicating the microstructure and dynamics of the two bilayers to be almost equal. This result deserves some considerations. Indeed, from a physico-chemical viewpoint, the two considered lipid mixtures are expected to form bilayers with distinct features. DOPE/DOPG (80/20) present a surface negative charge of 10 per 100 acyl tails; all the lipids are di-chained and, furthermore, all the acyl chains are mono-unsaturated. On the other hand, DOPE/DOPG/CL (65/23/12) present a surface negative charge of 21 per 100 acyl tails; CL is quadruple-chained, and, furthermore, around 20% of the acyl chains are di-unsaturated (the CL ones), while the remaining are mono-unsaturated. Thus, our results indicate the acyl chains organization within the bilayer to be scarcely affected by the surface charge, confirming what already observed for other lipids^36^. Moreover, we propose the increase of the cohesive interaction between the CL hydrocarbon chains due to the relatively small and rigid polar headgroup^33^,^36^, to be balanced by the disordering caused by the higher unsaturation degree of the CL acyl chains^33^.

In the presence of myxinidin or WMR at L/P = 30, the EPR spectra of the spin-labelled lipids in DOPE/DOPG bilayers do not change, see Fig. 3, as also confirmed by the parameters reported in Table 2. This implies that the interaction with the peptides, detected by the other experimental techniques, does not affect the average bilayer microstructure, suggesting that the peptides remains located at the membrane interface.

In the case of DOPE/DOPG/CL bilayers, a significant reduction of the 5-PCSL spectrum anisotropy is detected (highlighted by the dashed line in the Fig. 3) in the presence of myxinidin. This also reflects in the lower S and higher 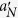 values and indicates that the peptide interaction disarrange the acyl chains packing favoring water molecules penetration in the bilayer. The perturbation is limited to chain segments close to the lipid headgroups, since 14-PCSL spectrum remains unperturbed (spectra not shown, see Table 2 for the 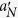 and *S* values).

WMR seems to be less effective in perturbing the 5-PCSL spectrum in DOPE/DOPG/CL bilayers. This evidence is in contrast with the results of other experimental techniques showing an enhanced interaction in the case of WMR with bilayers including CL in the lipid composition. A possible explanation is that WMR could preferentially interact with CL, inducing its segregation from the other lipids. 5-PCSL possibly remains in DOPE/DOPG-enriched domains, thus being poorly sensitive to the interaction. The bilayers response to the interaction with WMR was also investigated at higher peptide content. For both lipid systems, at L/P = 10 the EPR spectra show weaker signals suggesting strong re-arrangements of the bilayer microstructure. The 5-PCSL spectra are more anisotropic, as also reflected in the higher *S* values. In the case of DOPE/DOPG bilayers, the 14-PCSL spectrum is unaffected by the peptide even at this low L/P ratio, indicating that the peptide remains at the bilayer surface. Interestingly, for DOPE/DOPG/CL bilayers, the 14-PCSL spectrum shows an additional component (see outer wings of the spectrum highlighted by the arrows in Fig. 3), corresponding to spin-labelled lipid chains whose motion is restricted. This is a feature usually encountered for peptides that deeply insert in the lipid bilayer^32^. The slow motional component can be ascribed to spin-labelled acyl chains directly interacting with WMR. In contrast, the addition of myxinidin to both bilayers at L/P = 10 does not causes additional changes in the spectral features (spectra not shown, see Table 2 for the parameters). This result is in agreement with DLS experiments showing that high peptide concentrations do not induce fusion phenomena.

Preferential interaction of WMR and, to a minor extent, of myxinidin with DOPE/DOPG/CL bilayers can be ascribed to their higher surface charge; the electrostatic effect is further enhanced by the peculiar molecular architecture of CL^33^. The restrictions placed on the flexibility and re-orentational mobility of its polar headgroup by the tethering of the two phosphatidate moieties to a single glycerol molecule, reduce the capacity for steric selfshielding of the charged groups, which are both fully ionized^34^. Consequently, they are more “exposed” to interaction with the solvent and entities dissolved therein (e.g., peptides).

### Differential Scanning Calorimetry

**Figure 4:**
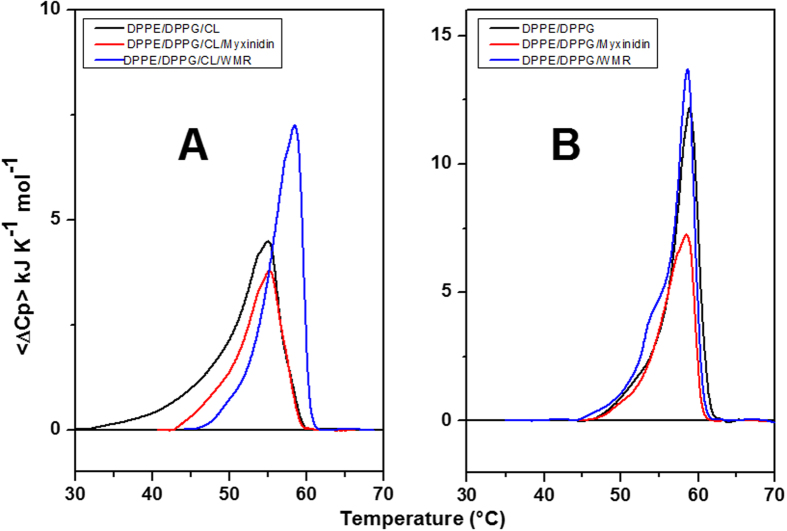
DSC profiles of DPPE/DPPG/CL (**A**) and DPPE/DPPG (**B**) liposomes in the absence (black line) and presence of myxinidin (red line) and WMR (blue line) at L/P = 10 ratio.

DSC experiments were performed to investigate the effect of peptides on the thermotropic phase transition of liposomes. Indeed, the effect of peptide binding on the thermodynamic parameters of the liposomes main transition allow to understand the potential tendency of the peptides to interact with lipid head groups and/or to perturb the packing of the lipid acyl chains^37^,^38^,^39^. In these experiments, the lipids DPPE and DPPG, which have the main phase transition in a temperature range suitable for DSC (63 °C and 41 °C for DPPE and DPPG, respectively), replace DOPE and DOPG in the liposomes, which show the main phase transition at about −16 °C. However, the replacement does not change the lipid head groups. Thus, DSC experiments were performed with MLVs formed by DPPE/DPPG/CL (65/23/12) and DPPE/DPPG (80/20). The DSC profiles of liposomes in the absence and presence of peptides are reported in Fig. 4.

The DSC curve of DPPE/DPPG (Fig. 4, panel B) shows a sharp transition centered at 59.0 ± 0.5 °C consistent with the lamellar gel (*L*_β_) to the liquid-crystalline (*L*_α_) phase transition according to literature data^40^. The DSC profile of DPPE/DPPG/CL (Fig. 4, panel A) shows a broader and less cooperative transition centered at 55.1 ± 0.5 °C compared to DPPE/DPPG (Fig. 4, panel B) liposomes. In order to study the perturbation effect of peptides on the phase transition of both liposomes, DSC experiments were performed at high peptide concentration (L/P = 10). DSC profiles of liposomes in the presence of myxinidin and WMR at L/P = 10 are shown in Fig. 4 and the corresponding thermodynamic parameters are collected in Table 3.

DSC results show that myxinidin marginally changes the thermodynamic parameters of the DPPE/DPPG/CL transition (Fig. 4, panel A and Table 3), whereas has a major effect on the DPPE/DPPG transition (Fig. 4, panel B and Table 3). In this case, myxinidin decreases the melting enthalpy of the DPPE/DPPG transition, without changing the overall shape of the DSC profile, thus suggesting a decrease in the acyl chain packing in the presence of the peptide^38^. On the contrary, WMR has a major effect on the CL containing liposomes. In particular, in the presence of WMR, the DPPE/DPPG/CL transition enthalpy increases (Table 3) and the main transition peak appears sharper and shifted at higher temperature (Fig. 4, panel A). This behavior is consistent with a preferential interaction of WMR with the negatively charged lipids DPPG and CL, leading to the clustering of these lipids and to the formation of DPPE-enriched domain, that melts more cooperatively and at higher temperature^41^. Interestingly, the DSC profile of the DPPE/DPPG/CL in the presence of WMR (Fig. 4, panel A) closely resembles that of the DPPE/DPPG liposomes (Fig. 4, panel B), suggesting that WMR preferentially segregates more CL than DPPG, leaving the remaining liposome domains enriched with DPPE/DPPG. Further, in the absence of CL, WMR marginally changes the thermodynamic parameters of the DPPE/DPPG transition (Fig. 4, panel B and Table 3). In particular, addition of WMR to DPPE/DPPG liposomes results in an enthalpy increase and in the appearing of a small shoulder in DSC profile at lower temperature. The absence of a shift to higher temperature of the melting transition suggests that WMR does not segregate DPPG as it does with CL. The slight modification of the DSC profile could be rather attributed to the change in the distribution of the dimension of the liposomes due to the fusion process induced by WMR binding (see *Lipid mixing assays and DLS results*). In conclusion, DSC results show that myxinidin perturbation effect on lipid bilayers is stronger in absence of CL. On the contrary, WMR is able to induce a higher degree of perturbation in the lipid bilayers containing CL. In particular, it induces the segregation of the anionic lipids CL and DPPG in DPPE/DPPG/CL liposomes. This ability of WMR to induce domains formation could play a key role in destabilizing the membranes and in promoting the antimicrobial activity. Further, we suppose that WMR preferentially segregates CL than DPPG and this hypothesis is supported by the absence of segregation phenomena in the membrane without CL. In fact, in DPPE/DPPG liposomes, no segregation of anionic DPPG is observed, supporting that the CL is a key element to determine the WMR-membrane interaction.

### Isothermal Titration Calorimetry

**Figure 5:**
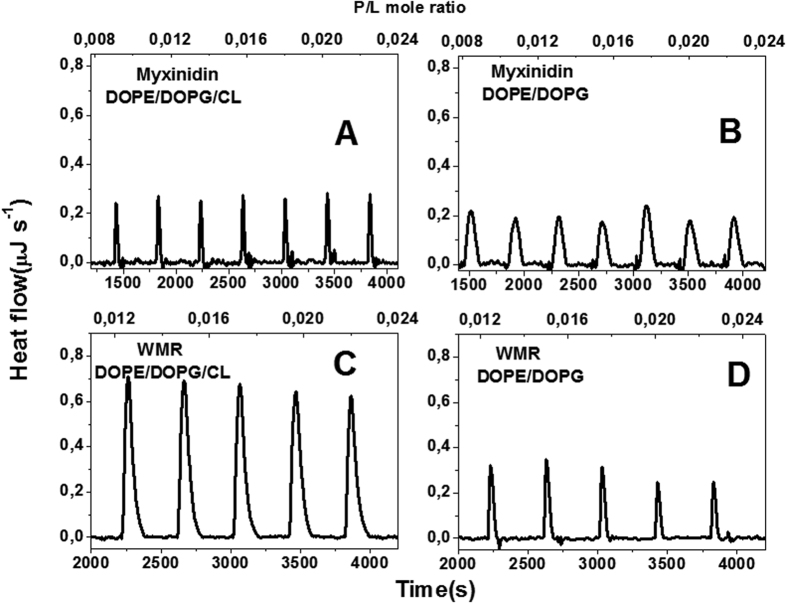
ITC traces obtained from the titration of DOPE/DOPG/CL (**A** and **C**) and DOPE/DOPG (**B** and **D**) liposomes with myxinidin (**A** and **B**) and WMR (**C** and **D**). All experiments were carried out at 25 °C in PBS buffer pH = 7.4.

The interaction between the peptides and the two studied membranes was further analyzed by means of isothermal titration calorimetry (ITC). The binding enthalpy for all the studied systems was directly measured by injecting a dilute peptide solution into the ITC cell containing a concentrated vesicles suspension (peptide-into-lipid titration). Under these conditions, the lipid is much in excess over the peptide during the whole titration experiment, and the injected peptide is completely bound to the membrane surface^42^,^43^. ITC traces obtained by the titration of the myxinidin and WMR into the liposomes in these conditions are reported in Fig. 5. The dilution of the peptides into buffer were also performed as control experiments (Fig. S1). As expected, in this condition of high (L/P) ratio, each injection produces the same heat. Analysis of the ITC traces obtained by the titration of myxinidin into DOPE/DOPG/CL (Fig. 5, panel A) and DOPE/DOPG (Fig. 5, panel B) liposomes provides negative values of association enthalpy of −5.9 ± 1.1 kJ/mol and −11.9 ± 1.3 kJ/mol respectively, revealing a favorable enthalpy contribution in the peptide-lipid interaction. The titration of WMR into DOPE/DOPG/CL (Fig. 5, panel C) and DOPE/DOPG liposomes (Fig. 5, panel D) gives negative values of association enthalpy of −38.6 ± 2.0 kJ/mol and −13.7 ± 0.7 kJ/mol, respectively, suggesting that the enthalpy contribution to the interaction is greater than for myxinidin. Since at this low peptide concentration the contribution of the peptide-peptide interaction can be excluded is likely that the difference in the interaction enthalpies are attributable to the difference in the peptide-lipid interactions. The higher binding enthalpy values of WMR could be due to the presence of three additional arginine and a tryptophan residues that are able to establish a variety of additional interactions (electrostatic, hydrogen bond) with negatively charged membranes. Since many AMPs exert their antimicrobial activity above a threshold L/P ratio^44^ due to the occurrence of peptide-peptide interaction, additional ITC experiments were performed with further increasing peptide concentration (low L/P ratio). In these conditions, ITC data reveal that the association enthalpy of myxinidin with the DOPE/DOPG/CL liposomes (Fig. S2, panel A) is very similar to the one obtained at low peptide concentration (Fig. 5, panel A), suggesting that no peptide-peptide aggregation and/or drastic change of lipid distribution occur. On the contrary, the ITC trace of the WMR titration into the DOPE/DOPG/CL liposomes at low L/P ratio was completely different (Fig. S2, panel C) from the one obtained at high L/P ratio (Fig. 5, panel C), suggesting the presence of strong peptide-peptide interaction and/or membrane reorganization above a threshold concentration. Similarly, ITC traces for the titration of myxinidin and WMR in the DOPE/DOPG liposomes at low L/P ratio (Fig. S2, panels B,D) are more complex with respect to the one observed at high L/P ratio (Fig. 5, panels B,D) confirming that both peptides are able to induce additional phenomena (as peptide aggregation and/or lipids reorganization) above a threshold concentration. Finally, reverse ITC experiments in which the lipid were injected into peptide solution were also performed. Analysis of the obtained binding curves for titration of myxinidin with DOPE/DOPG/CL and DOPE/DOPG provided a binding constant of 3.5·10^4^ M^−1^ and 1.8·10^4^ M^−1^, respectively and stoichiometry values in the range 15–17 lipids per peptide (Figs S3 and S4). The high stoichiometry values are in agreement with a surface binding^35^. Unfortunately, for WMR we were not able to obtain well reproducible and analyzable binding curves, this was expected as WMR induces fusion of liposomes at low L/P ratio (at the first injections in the titration experiments).

### Surface Plasmon Resonance

**Figure 6:**
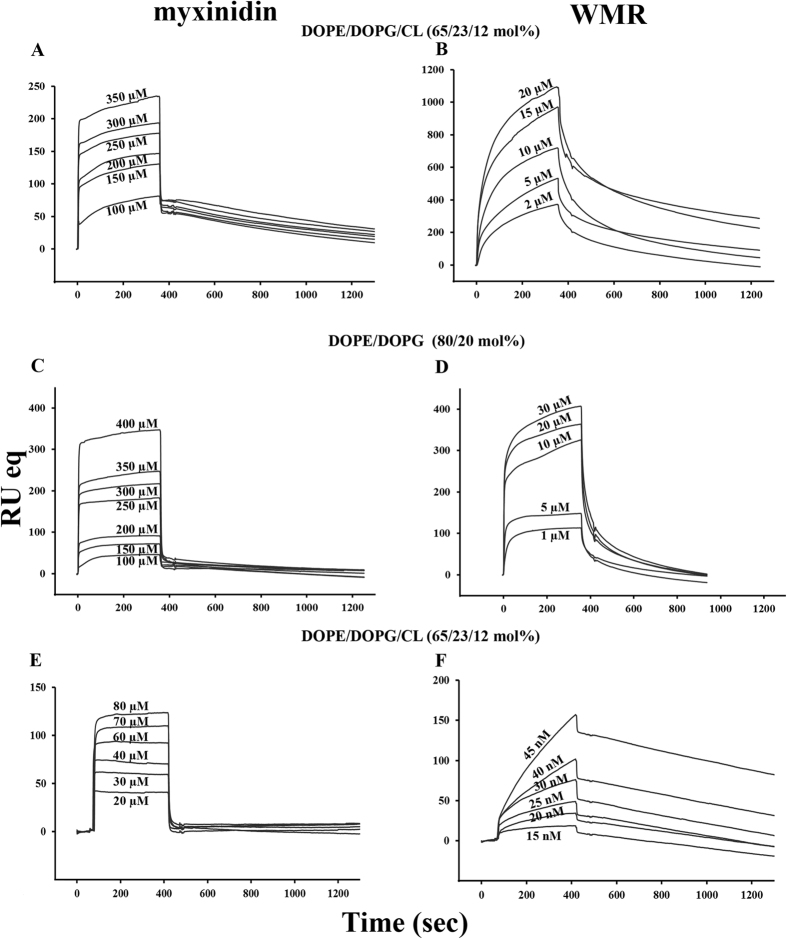
For myxinidin HPA sensorgrams in DOPE/DOPG/CL (65/23/12% mol) (**A**), in DOPE/DOPG (80/20% mol) (**C**) liposomes and L1 sensorgram in DOPE/DOPG/CL (65/23/12% mol) liposomes (**E**) at different peptide concentrations; for WMR, HPA sensorgrams in DOPE/DOPG/CL (65/23/12% mol) (**B**), in DOPE/DOPG (80/20% mol) (**D**) liposomes and L1 sensorgram in DOPE/DOPG/CL (65/23/12% mol) (**F**) liposomes at different peptide concentrations.

Monolayers of DOPE/DOPG/CL and DOPE/DOPG were absorbed onto the HPA and the L1 chip. Sensorgrams of the binding of myxinidin and WMR are shown in Fig. 6. The data for the interaction of both peptides with the L1 chip and DOPE/DOPG were not reproducible and are thus not reported. The RU signal intensity increased as a function of the peptide concentration. Most of the sensorgrams at higher concentrations did not return to zero, indicating that the peptides remained significantly bound to the surface or inserted into the hybrid bilayer membrane.

We used numerical integration analysis that uses nonlinear analysis to fit an integrated rate equation directly to a set of sensorgrams with different peptide concentrations^45^. Initially, we fitted the sensorgrams globally with the simplest 1:1 Langmuir binding model, and we obtained a poor fit (χ^2^ > 100), demonstrating that this model does not represent the lipid binding mechanism. A significantly improved fit was obtained when using the two-state reaction model, suggesting that there are likely to be at least two steps involved in the interaction between both peptides and the two hybrid bilayer membranes. The first step corresponds to the actual binding of the peptide to the surface, while the second step is the insertion of the peptide into the hydrophobic core of the membrane, and its rearrangements inside the membrane bilayer. The average values for the rate constants obtained from the two-state model analysis are listed in Table 4 along with the affinity constant values (K_A_).

Analysis of the data reported allowed several important observations. The first interesting observation is that notwithstanding the different values obtained for the two peptides in the different membranes and on different chips, the obtained K_A_ are in the typical range of peptides that strongly interact with membrane bilayers. Moreover, we obtained a different behavior for the two peptides, with the affinity constants of myxinidin always lower than those of WMR. Moreover, the affinity of WMR for DOPE/DOPG/CL membranes is higher when compared to DOPE/DOPG. We further analyzed the K_1_ and K_2_ of the two steps for the monolayer; the obtained results indicated that the first step corresponding to the charge interaction is always stronger that the second step that corresponds to the rearrangements inside the membrane bilayer. The K_1_ values of WMR for DOPE/DOPG/CL membranes is very high, confirming the results obtained by the other experimental techniques that indicate a stronger interaction of WMR with CL containing liposomes. The K_2_ values are generally lower but the highest is again obtained with WMR and DOPE/DOPG/CL membranes indicating that also the complex events that take place inside the membrane are more significant for WMR. The analysis of the K for the interaction of both peptides with the L1 chip and DOPE/DOPG/CL membrane, clearly show again a stronger interaction for WMR compared to myxinidin. A deeper analysis of the K_1_ and K_2_ shows that K_2_ is significantly higher, which may be attributed to the conformational rearrangements taking place inside the bilayer which may involve the strong interaction with CL. The ratio K_A bilayers_/K_A monolayer_ demonstrates that the binding to bilayers is greater than to monolayers for WMR (K_A bilayers_/K_A monolayer_ = 2.4) indicating that the peptide is influenced by the membrane’s inner leaflet and clearly discriminating the pore formation ability of WMR from other membrane insertion mechanisms that may involve myxinidin (K_A bilayers_/K_A monolayer_ = 0.19).

Interestingly, the affinity constant of myxinidin for DOPE/DOPG/CL membrane is higher compared to DOPE/DOPG membrane (see data for the HPA chip), this result strongly suggest that the lower biological activity of myxinidin against *P. aeruginosa* is not attributable to a weak binding to the bacteria membrane but rather to a myxinidin incapacity to affect the membrane stability. All our data confirm this hypothesis showing that myxinidin binding to DOPE/DOPG/CL is on the membrane surface and do not determine drastic change of lipid distribution or fusion phenomena which is further confirmed by the analysis of the ratio K_A bilayers_/K_A monolayer_.

## Conclusions

The goal of AMP development is to understand and optimize biophysical parameters, minimize eukaryotic cell toxicity, and maximize antimicrobial activity. In previous papers we showed that the simultaneous substitutions of residues present in positions 3, 4, and 11 with arginine and the addition of a tryptophan at the N terminus (WMR) of the native sequence of myxinidin resulted in a significant increase in activity^8^,^9^. The previously reported data on the antibacterial activity of myxinidin and WMR showed that the latter is more effective with all tested bacteria, while myxinidin showed a lower activity for some bacteria as *P. aeruginosa*. The analysis of the membrane composition pinpoints the fact that *Gram-negative* bacteria contain PE, PG, and CL, and a deeper analysis shows that *P. aeruginosa* contains a high percentage of the negatively charged CL. We previously speculated that the obtained biological results might be correlated to the greater susceptibility to charged residues of *P. aeruginosa.* To find a correlation between the WMR and myxinidin biological activity and the membrane composition of the bacteria we performed a complete biophysical characterization of the interaction of these two peptides with two liposomes mimicking the *E. coli* and *P. aeruginosa* membranes, and we found that the effects of the two peptides are significantly different. All the results obtained by different experimental techniques have coherently shown that, even though both myxinidin and WMR interact with the two membranes (high affinity constants), their effect on membrane microstructure and stability are different. In particular, myxinidin does not perturb significantly the CL containing membrane as shown by DSC, EPR, ITC and fluorescence results. These findings allow attributing the lower biological activity of myxinidin against *P. aeruginosa* to its incapacity to affect the membrane stability. On the contrary, myxinidin has a major perturbation effect on the membrane containing a minor percentage of negatively charged lipids, causing a lower lipid acyl chain packing in the membrane as shown by DSC and EPR results and probably facilitating the formation of transient pore as suggested by leakage experiments. On the other hand, our data show that WMR has high affinity constants for both membranes (as shown by SPR results) combined with a great capacity to drastically perturbing the membranes properties above a peptide concentration threshold. Particularly, DLS and lipid mixing assays results clearly show that WMR induce membrane fusion processes in both DOPE/DOPG/CL and DOPE/DOPG liposomes. These findings are perfectly coherent with the high antimicrobial activity of WMR compared with myxinidin. Further, DSC show that WMR induces the segregation of CL away from zwitterionic lipids and probably this segregation favors membrane destabilization introducing phase boundary defects between the lipid domains. It is interesting to speculate that the formation of domains may potentially contribute to the antibacterial activity of AMPs^46^,^47^. Although a direct correlation between the observed lipid domains and the antimicrobial activity of the peptides still needs to be established, our results point to the key role played by this phenomenon.

In summary, the role of lipid composition on the antimicrobial activity of myxinidin and WMR has been dissected. The presence of the anionic CL has been demonstrated to be a key element in the peptide ability to bind and perturb the membrane. Our data are able to correlate the observed antimicrobical activity of these peptides with a distinct behavior at the level of peptide-lipid interactions. These detailed information represent a prerequisite for the design of novel AMPs with improved activity and able to discriminate between membranes.

## Additional Information

**How to cite this article:** Lombardi, L. *et al*. Antimicrobial peptides at work: interaction of myxinidin and its mutant WMR with lipid bilayers mimicking the P. aeruginosa and *E. coli* membranes. *Sci. Rep.* **7**, 44425; doi: 10.1038/srep44425 (2017).

**Publisher's note:** Springer Nature remains neutral with regard to jurisdictional claims in published maps and institutional affiliations.

## Supplementary Material

Supplementary Material

## Figures and Tables

**Table 1:** Physico-chemical characteristics of DOPE/DOPG and DOPE/DOPG/CL liposomes in the presence and absence of myxinidin or WMR peptides at the indicated lipid/peptide (L/P) ratio.

		*<D> (cm*^*2*^*/sec)*	*R*_*h*_ *(nm)*
*DOPE/DOPG*		(2.6 ± 0.3) 10^−8^	95 ± 9
	+myxinidin (L/P = 10)	(2.7 ± 0.3) 10^−8^	90 ± 5
	+WMR (L/P = 30)	(5.30 ± 0.9) 10^−9^	460 ± 80
	+WMR (L/P = 10)	—^a^	—^a^
*DOPE/DOPG/CL*		(2.8 ± 0.2) 10^−8^	88 ± 5
	+myxinidin (L/P = 10)	(2.9 ± 0.3) 10^−8^	80 ± 9
	+WMR (L/P = 30)	(2.4 ± 0.5) 10^−8^	104 ± 2
	+WMR (L/P = 10)	(5.5 ± 0.2) 10^−9^ ^b^	440 ± 120^b^
		(2.6 ± 0.3) 10^−8^ ^c^	96 ± 10^c^

^a^Data not shown for a bad correlation function; ^b^first peak; ^c^second peak.

**Table 2:** Hyperfine coupling constant, 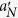 , and order parameter, *S*, of *n*-PCSL in DOPE/DOPG and DOPE/DOPG/CL liposomes, in the absence and presence of myxinidin or WMR^a^.

		5-PCSL		14-PCSL	
		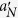 */G*	*S*	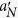 */G*	*S*
*DOPE/DOPG*		15.2	0.62	13. 9	0.21
	+myxinidin (L/P = 30)	15.3	0.60	14.0	0.19
	+myxinidin (L/P = 10)	15.2	0.61	14.1	0.20
	+WMR (L/P = 30)	15.1	0.62	14.0	0.20
	+WMR (L/P = 10)	15.2	0.67	14.2	0.21
*DOPE/DOPG/CL*		15.2	0.61	14.0	0.20
	+myxinidin L/P = 30)	15.6	0.54	13.9	0.19
	+myxinidin L/P = 10)	15.5	0.53	14.0	0.19
	+WMR (L/P = 30)	15.2	0.58	14.1	0.20
	+WMR (L/P = 10)	14.9	0.71	14.1^b^	0.23^b^

^a^The estimated error in 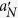 is ± 0.2 G; in *S*, <±4%; ^b^Determined from the prevalent faster component.

**Table 3:** Thermodynamic parameters obtained from the DSC profiles of DPPE/DPPG and DPPE/DPPG/CL liposomes in the absence and presence of myxinidin and WMR at lipid/peptide ratio (L/P) = 10.

		*Δ*_*m*_*H (kJ/mol*)^a^	*T*_*m*_ (*°C*)
*DPPE/DPPG*		57.4 ± 2.5	59.0 ± 0.5
	+myxinidin	39.1 ± 3.0	58.5 ± 0.5
	+WMR	61.6 ± 3.0	58.7 ± 0.5
*DPPE/DPPG/CL*		31.1 ± 3.0	55.1 ± 0.5
	+myxinidin	26.0 ± 2.5	55.2 ± 0.5
	+WMR	50.3 ± 3.0	57.1 ± 0.5

^a^Values were normalized against total lipid moles.

**Table 4:** Association (ka_1_, ka_2_) and dissociation (kd_1_, kd_2_) rate constants obtained for the HPA and the L1 chip using the two state model.

*HPA chip*							
	*k*_*a1*_	*k*_*d1*_	*K*_*1*_	*k*_*a2*_	*k*_*d2*_	*K*_*2*_	*K*_*A*_
DOPE/DOPG							
myxinidin	(3.55 ± 0.12)·10^1^	(3.19 ± 0.14)·10^−2^	1.1·10^3^	(8.34 ± 0.29)·10^−3^	(1.63 ± 0.03)·10^−3^	5.1	5.61·10^3^
WMR	(7.45 ± 0.01)·10^2^	(3.55 ± 0.17)·10^−2^	2.1·10^4^	(7.94 ± 0.29)·10^−3^	(5.13 ± 0.12)·10^−3^	1.5	3.15·10^4^
DOPE/DOPG/CL							
myxinidin	(6.64 ± 0.18)·10^2^	(1.33 ± 0.04)·10^−1^	5.0·10^3^	(5.92 ± 0.08)·10^−3^	(1.12 ± 0.03)·10^−3^	5.3	2.65·10^4^
WMR	(5.57 ± 0.02)·10^2^	(1.47 ± 0.03)·10^−2^	3.8·10^4^	(4.47 ± 0.05)·10^−3^	(2.33 ± 0.09)·10^−3^	1.9	7.22·10^4^
***L1 chip***							
DOPE/DOPG/CL							
myxinidin	(9.43 ± 0.19)·10^2^	(5.17 ± 0.02)·10^−1^	1.8·10^3^	(2.13 ± 0.09)·10^−3^	(1.15 ± 0.03)·10^−3^	1.8	5.19·10^3^
WMR	(6.98 ± 0.05)·10^2^	(3.53 ± 0.03)·10^−1^	2.0·10^3^	(2.85 ± 0.04)·10^−2^	(3.25 ± 0.05)·10^−4^	88	1.75·10^5^

The affinity constants K_1_ and K_2_ are for the first (K_1_ = k_a1_/k_d1_) and for the second (K_2_ = k_a2_/k_d2_) steps respectively, and the affinity constant (K_A_) determined as (k_a1_/k_d1_)·(k_a2_/k_d2_) is for the complete binding process.
