# Supplementary material for: Antimicrobial peptides at work: interaction of myxinidin and its mutant WMR with lipid bilayers mimicking the P. aeruginosa and E. coli membranes
Source: Sci Rep. 2017 Mar 15;7:44425. doi: 10.1038/srep44425 (PMC5353584; doi:10.1038/srep44425)
Supplement: Supplementary Material [file srep44425-s1.pdf]

Antimicrobial peptides at work: interaction of myxinidin and its mutant WMR with lipid bilayers mimicking the *P. aeruginosa* and *E. coli* membranes

Lucia Lombardi[1#] Marco Ignazio Stellato[2#], Rosario Oliva[2], Annarita Falanga[3], Massimiliano Galdiero[1], Luigi Petraccone[1], Geradino D'Errico[2], Augusta De Santis[2], Stefania Galdiero[3], Pompea Del Vecchio[2*]

[1]Department of Experimental Medicine, Second University of Naples, via De Crecchio, 80134 Naples, Italy
[2]Department of Chemical Sciences, University of Naples "Federico II", via Cintia, 80126 Naples, Italy
[3]Department of Pharmacy, University of Naples "Federico II", Via Mezzocannone 16, 80134 Naples, Italy

[#]these authors contributed equally.
*to whom correspondence should be addressed.
Tel: +39 081 674255; Fax: +39 081 674090; e-mail : pompea.delvecchio@unina.it

Supplementary material

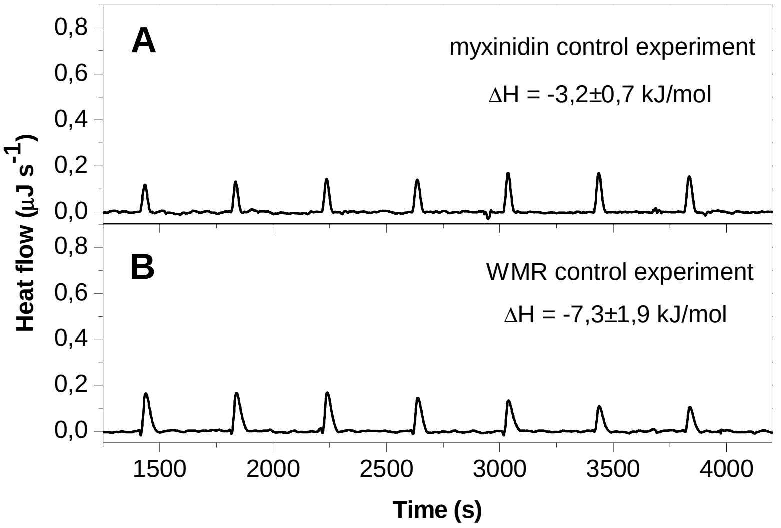

Fig. S1 ITC traces obtained from the dilution of (A) myxindin and (B) WMR in PBS buffer. The enthalpy changes of dilution of two peptides are reported in the figure. The experiments were performed at the temperature of 25 °C.

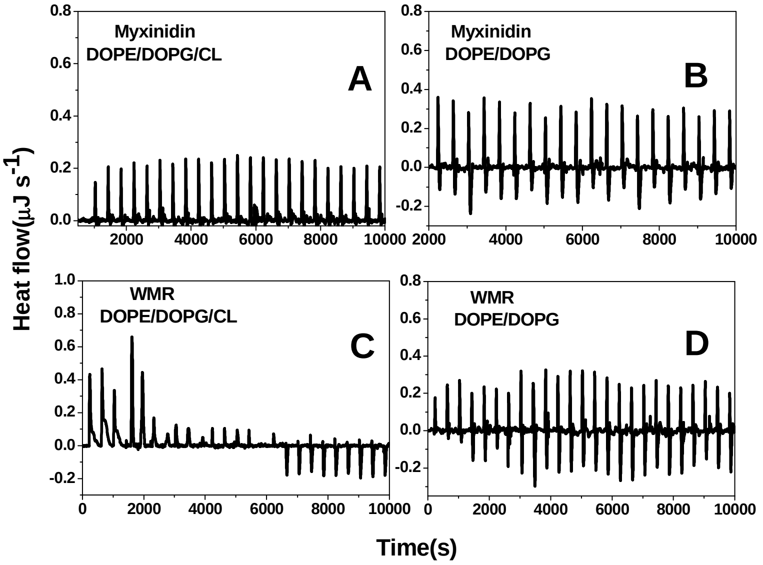

Fig. S2 ITC traces obtained from the titration of DOPE/DOPG/CL (A and C) and DOPE/DOPG (B and D) liposomes with myxinidin (A and B) and WMR (C and D) at high peptide concentrations (up to L/P=10). All experiments were carried out at 25 °C in PBS buffer pH 7.4.

ITC measurements (lipid-into-peptide titrations)

In order to determine the apparent bindind constant (K_{app}) and the stoichiometry (n, number of lipid molecules bound per peptide molecule), a lipid-into-peptide titrations were performed using the same apparatus as described in the *Methods* section. A peptide solution (50-70 µM) was placed in the calorimeter cell (~1 mL) and a lipid dispersion (12-20 mM) was injected in aliquots of 10 µL with 400 s intervals between each injections (total number of injections: 25). The heat peaks recorded after the system reached saturation were used to correct for heat of dilution of lipid dispersion. The integrated raw data, normalized for the concentration of added lipids, were reported as a function of L/P mole ratio and analyzed using a nonlinear least-squares minimization algorithm to a theoretical independent binding sites isotherm. Since the binding of peptide is limited to the external lipid leaflet, a correction factor of γ = 0,5 was applied to the lipid concentration (Seelig, J. *Biochim. Biophys. Acta* (1997) 1331, 103-116).

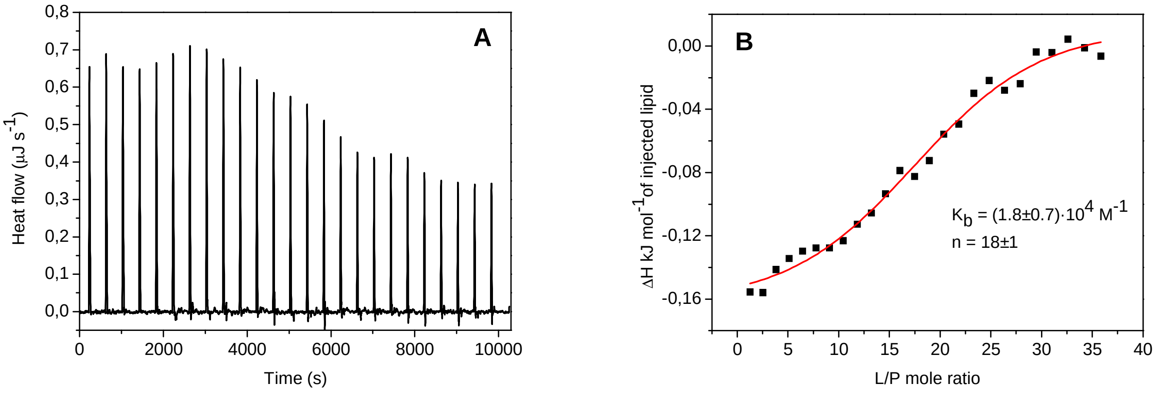

Fig. S3 (A) ITC trace obtained from the titration of 12 mM DOPE/DOPG large unilamellar vesicles with a myxinidin solution of 50 µM. (B) Integrated heats of binding plotted as a function of L/P ratio. The red solid line represents the best curve fit. The experiment was performed at 25 °C in PBS buffer, pH 7.4.

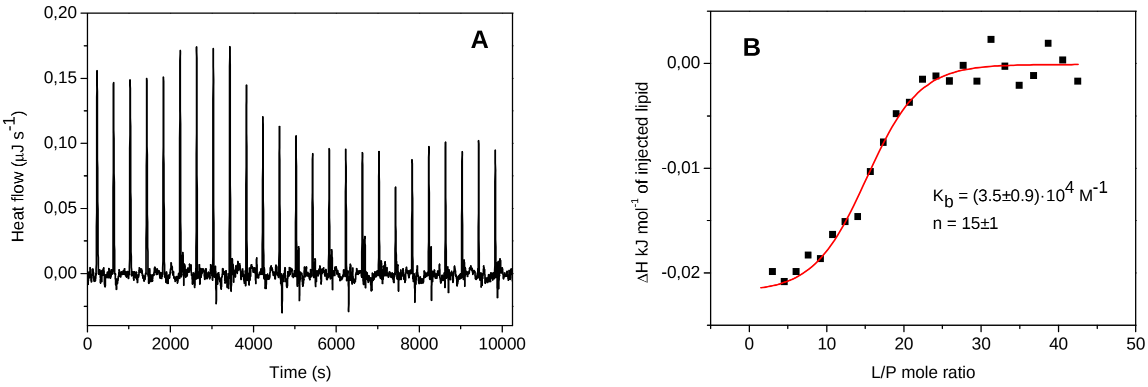

Fig. S4 (A) ITC trace obtained from the titration of 20 mM DOPE/DOPG/CL large unilamellar vesicles with a myxinidin solution of 70 µM. (B) Integrated heats of binding plotted as a function of L/P ratio. The red solid line represent the best curve fit. The experiment was performed at 25 °C in PBS buffer, pH 7.4.
